# Facilitators, barriers and support needs to GLA:D exercise adherence – a mixed method study

**DOI:** 10.1186/s13102-024-00913-6

**Published:** 2024-06-13

**Authors:** Franziska Matile, Irina Nast, Karin Niedermann

**Affiliations:** ZHAW School of Health Professions, Institute of Physiotherapy, Zurich University of Applied Sciences, Katharina-Sulzer-Platz 9, Winterthur, 8401 Switzerland

**Keywords:** Osteoarthritis, Physical activity, Exercise therapy, Adherence, Barriers, Facilitators

## Abstract

**Background:**

Knee and hip osteoarthritis (OA) are among the most common musculoskeletal joint diseases worldwide. International guidelines recommend exercise and education as first-line interventions for their management. The Good Life with osteoArthritis Denmark (GLA:D) programme aims to achieve self-management using group exercise and education sessions. It also encourages participants to stay physically active and perform GLA:D exercises (GE) twice weekly after programme end. This study investigated the participants’ self-reported level of physical activity (PA) and self-reported adherence to the GE between five and 17 months after completion of the GLA:D programme and also explored the barriers, facilitators and support needs to achieve long-term adherence to GE.

**Methods:**

A mixed method study using an exploratory sequential design was performed. A qualitative phase, involving semi-structured interviews and a focus group, led to the development of a questionnaire on participants’ level of PA, as well as ratings of the barriers, facilitators and support needs for the achievement of long-term adherence to GE. In a second quantitative phase, the survey was conducted online with former GLA:D participants from Switzerland. Descriptive statistical analysis and a group comparison between adherent and non-adherent participants to the GE were performed using Fisher’s exact test, odds ratio, and confidence interval.

**Results:**

Eleven former GLA:D participants attended the interviews and focus group, and former GLA:D participants (30% response rate) participated in the survey. Of these, 84% (*n* = 285) reported to reach the recommended level of PA and 53% (*n* = 178) GE adherence. The top barrier to GE adherence was *no/little self-discipline to perform GE* (40%, *n* = 112) and the top facilitator was *GE are easy to perform* (93%, *n* = 300). The top 3 items regarding support needs to enhance GE adherence were *a shortened version (max. 30 min) of the GE home programme* (75%, *n* = 255), *monthly continuation of small GE groups under GLA:D physiotherapists’ supervision* (65%, *n* = 221), and *monitoring with regular testing of individual progress* (65%, *n* = 221).

**Conclusions:**

The top barriers and facilitators should be considered by those responsible for the GLA:D programme and may need to be specifically addressed during and after the programme. The development of a shortened version of the GLA:D programme, a post-GLA:D group, and monitoring with regular testing seem crucial for enhancing GE adherence.

**Clinical Trial Registration:**

not applicable.

**Supplementary Information:**

The online version contains supplementary material available at 10.1186/s13102-024-00913-6.

## Background

Osteoarthritis (OA) is the most common musculoskeletal joint disease worldwide [1]. A large proportion of the affected people suffer from OA of the weight-bearing joints, with knee OA and hip OA mostly common [2]. The most important symptoms are pain, impaired physical function and reduced quality of life [3]. Thus, OA not only has a significant negative impact on the affected individuals, but also on the health system due to high socio-economic costs [4]. The Global Burden of Disease Study 2017 revealed a global point prevalence of 3,754.2/100,000 and an annual incidence of 181.2/100,000 persons with OA across 195 countries [2]. The same study showed a 9.3% global rise in the prevalence of OA between 1990 and 2017 along with an increase in prevalence with age, confirming that elderly people are more often affected. With increasing life expectancy, a rise in future prevalence is very likely [2]. Effective OA management is of great importance in order to minimise the personal and socio-economic consequences of OA [4].

International clinical guidelines for the management of OA recommend exercise and education as first-line interventions [5, 6]. Exercise is a subset of physical activity (PA) that is “planned, structured and repetitive, and has as a final or an intermediate objective, the improvement or maintenance of physical fitness” [7]. It is important to provide education alongside exercise for people with knee and hip OA [8]. The World Health Organisation (WHO) 2020 guidelines for PA [9] recommend persons being physically active at moderate intensity for at least 150 to 300 min a week or, alternatively, at vigorous intensity for at least 75 to 150 min a week, or a combination of both intensities and a reduction in sedentary time. For additional health benefits, it is recommended to perform muscle-strengthening activities on two or more days a week.

The Good Life with osteoArthritis Denmark (GLA:D) programme was developed in Denmark in 2013 as an implementation of the international clinical guidelines for the management of knee and hip OA into clinical practice [5, 6]. In the GLA:D programme, GLA:D-certified physiotherapists provide two group patient education sessions and 12 exercise group sessions. Its aims are to relieve pain, improve physical function and quality of life, and promote self-management strategies to foster long-term adherence to PA and GE [10]. The GE programme includes 10 exercises that are standardised but indivdualised on four levels of progressive difficulty. Neuromuscular exercises (NEMEX) form the core of the GE, together with core strengthening and walking exercises. Each participant performs his/her individual exercise programme, which is regularly adapted to achieve progress. Participants are recommended to continue performing the GE at least twice weekly after completion of the programme. The GLA:D programme has been implemented in Switzerland since 2019. The results of the GLA:D programmes, both internationally [11] and in Switzerland [12], show remarkable improvements in pain, physical function and quality of life at programme end, which are sustained at the one-year follow-up. The knee pain was reduced by 27% directly after the programme and by 26% in the one-year follow-up. The knee physical function was improved by 16% directly after the programme and by 12% after one-year follow-up [12]. These results of an implementation (i.e. best practice) project are notable, since research usually shows that PA interventions for knee and hip OA are effective at improving outcomes only for a short period (≤ six months after intervention cessation) [13].

There is evidence that the majority of people with knee and hip OA are less active than healthy people [14] and that only a small to moderate proportion of these people meet the recommended level of PA [15]. The study by Pisters et al. showed that the recommended level of PA and exercise adherence declined 15 months after a PA-enhancing intervention [16], thus justifying the need for long-term support of PA and exercise adherence. Additionally, Pisters et al. found a positive relationship between adherence to PA and exercise and intervention outcomes in people with knee and hip OA [16]. The WHO defines adherence as “the extent to which a person’s behaviour […] corresponds with agreed recommendations from a health care provider” [17].

Investigation of barriers and facilitators is important in obtaining a better understanding of long-term adherence to GLA:D exercises. General barriers and facilitators of PA in people with knee and hip OA have already been the subject of research [18, 19]. In Kanavaki et al., the main barriers were found to be pain, physical limitations, negative PA experiences, lack of motivation and behavioural regulation, whereas the main facilitators identified were positive PA experience, knowledge, adjusting and prioritising PA and social support [19].

To date, little is known about the barriers, facilitators and support needs affecting adherence to PA and GE after the GLA:D programme, although it can be assumed that the GE and education programme reduces some of the barriers and strengthens some of the facilitators.

An understanding of the most important factors influencing long-term adherence to GE and the incorporation of effective interventions to support long-term adherence to GE are critical. Cinthuja et al. showed some effective strategies to improve long-term exercise adherence by people with lower limb OA, such as providing booster-sessions and telephone-linked communication [20].

This study aims to investigate GLA:D Switzerland participants’ self-reported level of PA and self-reported adherence to GE between five and 17 months after programme completion, as well as to explore the barriers, facilitators and support needs to achieve GE long-term adherence.

## Methods

### Study design

**Fig. 1:**
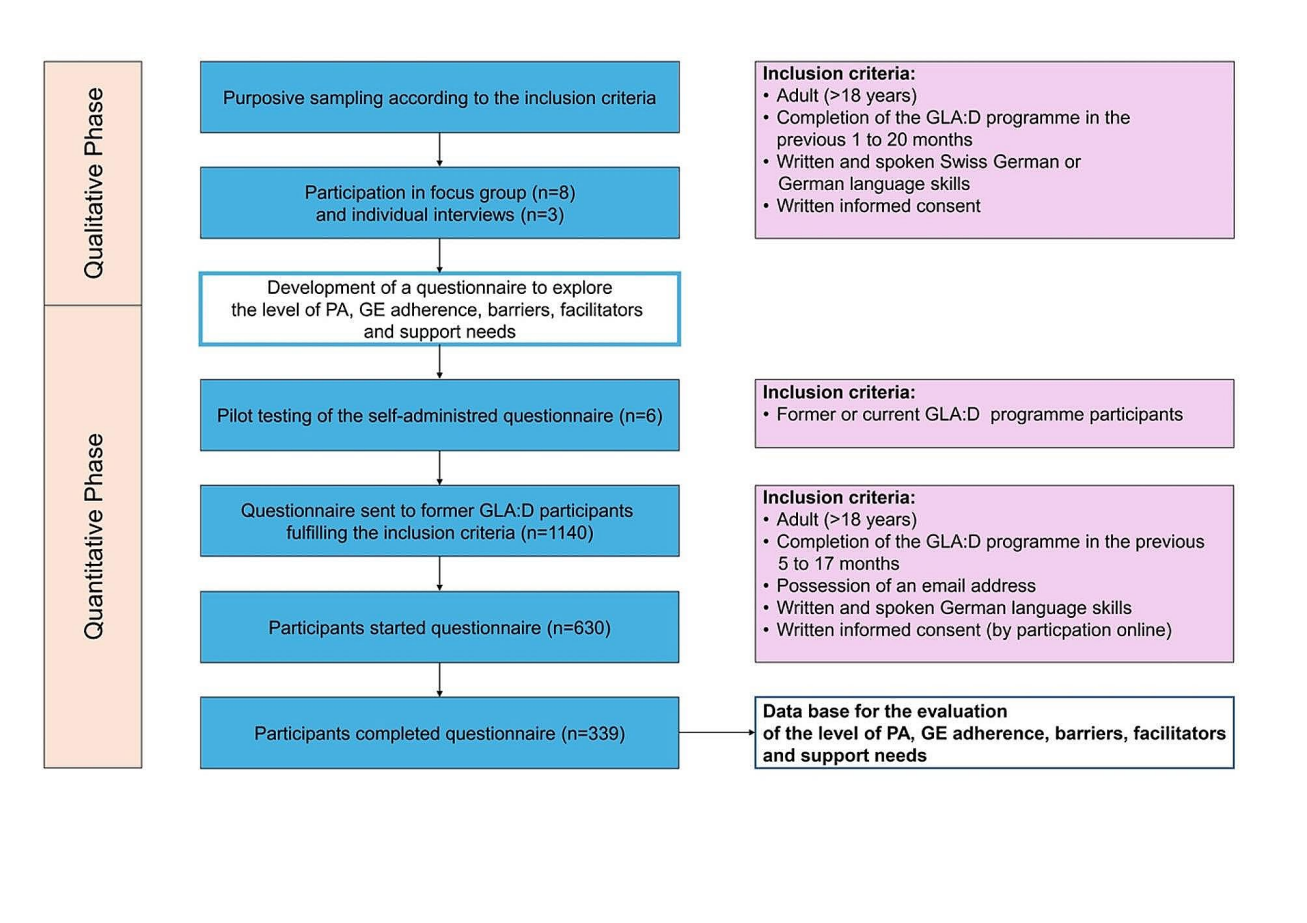
Mixed method study design flow chart

A mixed-methods study with an ‘exploratory sequential design’ was conducted [21]. The study was carried out in two phases: (1) a qualitative phase, during which semi-structured individual interviews and a focus group were carried out, serving as basis for (2) a quantitative phase, when an online survey was performed. More details to the mixed-methods study design can be found in the flow chart in Fig. 1.

### Qualitative phase

#### Setting and participants

The participants were recruited by certified GLA:D PTs from the surroundings of the University (within a radius of 25 km), by means of the purposive sampling method. Inclusion criteria were: (1) Adult (> 18 years); (2) Between 1 and 20 months after conclusion of the GLA:D programme; (3) written and spoken Swiss German or German language skills; and (4) written informed consent. Additionally, a maximum variation strategy was followed by the researchers when including participants by covering a broad range of demographic and disease-related characteristics in terms of age, gender, knee OA, hip OA and rural or urban living. Due to availability of the participants, participants were allocated either to the individual interview or to the focus group setting, depending on their time availability. Individual telephone interviews and a face-to-face focus group were conducted between August and October 2021, following the practical guide for focus groups by Krueger [22]. The individual interviews lasted between 25 and 30 min. The focus group took place at the Zurich University of Applied Sciences in Winterthur and was of 90 min duration. Two experienced physiotherapy researchers conducted the individual interviews (KN (*n* = 2), IN (*n* = 1)) and the focus group (KN, IN). The focus group was moderated by IN and KN took field notes. The individual interviews and the focus group were conducted in the Swiss German language and were audiotaped. As the interviews and focus group were originally developed in German, the materials were translated into English for the purpose of this publication. The language was translated with DeepL Translator and revised by a native speaker.

#### Interviews and focus group

In a first step, a question guide for the semi-structured interviews and the focus group was developed. The structure and the content of the question guide was based on expert opinion and a literature review [23]. The experts were a physiotherapist who has previously delivered the GLA:D programme and two researchers who have evaluated the impact and implementation of the GLA:D programme in Switzerland. The questions encompassed the three points of interest that were defined a priori: (1) Attitude towards PA and GE; (2) Barriers and facilitators affecting long-term adherence to PA and GE; and (3) Support needed to enhance long-term adherence to PA and GE. The semi-structured interview guide is shown in the Additional file 1.

#### Analysis

Transcription and Coding of the interviews and the focus group was conducted by the first author (FM) and advised by the two co-researchers (KN, IN). For the analysis the software of MAXQDA (Version 2020) was used. During transcription, the language was transliterated from Swiss German to German language. An inductive content analysis according to Elo and Kyngäs (2008) was performed. In the organising phase, the transcripts were open-coded and then condensed into items. In the grouping phase, the items were firstly allocated to subcategories, then to generic categories and finally to the main categories [24]. The category system is provided in the Additional file 2.

### Quantitative phase

#### Setting and participants

The study sample for the survey were former GLA:D participants from Switzerland. The inclusion criteria were: (1) completion of the GLA:D programme in the past 5 to 17 months; (2) Possession of an email address; and (3) written and spoken German language skills. Recruitment was supported by the management of GLA:D Switzerland, since the study participants were selected from the GLA:D Switzerland data register. The invitation links were sent by email to all 1,140 former GLA:D participants fulfilling the inclusion criteria. A reminder was sent after two weeks.

#### Questionnaire

The self-administered questionnaire contained questions on: (1) Demographic and disease-related characteristics; (2) The level of PA, using the German Short-Form International Physical Activity Questionnaire (IPAQ-SF) [25], which has acceptable measurement properties [26]; (3) Frequency and duration of self-reported GE adherence; (4) Barriers and facilitators to the recommended GE performance; and (5) Support needs. The questions on barriers and facilitators were derived from the qualitative interview and focus group data (see Additional file 2), as well as the findings on barriers and facilitators in knee and hip OA populations in the systematic review by Kanavaki et al. [19]. This resulted in 31 barrier and 31 facilitator items. Questions on the support needed to promote long-time adherence to GE were derived from the qualitative interview and focus group data and were also integrated into the questionnaire. Prior to commencing data collection, the comprehensibility of the online survey and its duration were pilot-tested on six former or current GLA:D programme participants. The feedback of these individuals was integrated into the final version of the questionnaire. The questionnaire was shortened and the wording of the questions improved.

Five-point scales were used for the rating of the barrier and facilitator items by the survey participants. Barriers: ‘not hindering at all’, ‘little hindering’, ‘rather hindering’, ‘very hindering’, ‘not applicable’; and Facilitators: ‘not facilitating at all’, ‘little facilitating’, ’rather facilitating’, ‘very facilitating’, ‘not applicable’. The usefulness of the support needs was rated on a 4-point scale (‘not useful at all’, ‘little useful’, ’rather useful’, ’very useful’) (see Additional file 3). The survey software Unipark was employed for the online survey (QuestBack, https://www.unipark.com). For purpose of this publication the questionnaire was translated into English.

### Statistical analysis

Demographic data is presented as absolute and relative frequencies or as mean values with standard deviations, as appropriate. The levels of PA and self-reported GE adherence, barriers, facilitators and support needs are all expressed as frequencies. To compare the GE-adherent participants (those performing the recommended GE ≥ 2 times per week) and the GE-non-adherent participants (those performing GE < 2 times per week), barriers and facilitators were rated separately, and the data analysed by group. The group differences were compared for the frequencies of barriers and facilitators, respectively, using the Fisher’s exact test. Therefore, the answer categories were collapsed into the two groups ‘not at all/little hindering/facilitating’ and ‘rather/very hindering/facilitating. The answer category ‘not applicable’ was excluded in the statistical analysis. The statistics revealed the odds ratio (OR) with the confidence interval (CI) and the *p*-value. The level of significance was set to *p* < 0.05. Data was exported and analysed by FM with the support of a statistician using the statistical software RStudio (Version 1.2.5019). The Tables were created with Microsoft Excel (Version 16.43).

## Results

### Participants

Eight persons (four females, 50%) with a mean age of 66 (±9.8) years participated in the focus group and three persons (two females, 66%) with a mean age of 60 (± 7.6) participated in the single interviews. From the sample of 1,140 persons, 630 people started the survey and 339 (30%) completed it. Participants were mostly female (n = 227, 67%) with a mean age of 67 (±9.3) years, ranging from 29 to 89 years. Table 1 provides a detailed overview of the participants’ demographic and disease-related characteristics for the qualitative and quantitative phase.

**Table 1:** Participant characteristics

Qualitative Phase			
	All participants	Single interview	Focus group
	(*n* = 11)	(*n* = 3)	(*n* = 8)
**Age, years, mean (SD)**	64 (± 9.3)	60 (± 7.6)	66 (± 9.8)
**Gender, n (%)**			
Women	6 (55)	2 (66)	4 (50)
Men	5 (45)	1 (33)	4 (50)
**Joint(s) with OA, n (%)**			
Knee(s)	9 (82)	3 (100)	6 (75)
Hip(s)	2 (18)	0	2 (25)
**Working, n (%)**			
Yes	6 (55)	2 (66)	4 (50)
No	5 (45)	1 (33)	4 (50)

Quantitative Phase			
	All participants	GE-adherent	GE-non-adherent
	(*n* = 339)	(*n* = 178 (53%)	(*n* = 161 (47%)
**Age, years, mean (**± **SD**)	67 (± 9.3)	68 (± 8.1)	65 (± 10.2)
**Women, n (%)**	227 (67)	120 (67)	107 (66)
**Highest level of education, n (%)**			
mandatory school completed	12 (4)	6 (3)	6 (4)
secondary level	181 (53)	103 (58)	78 (48)
tertiary level	146 (43)	69 (39)	77 (48)
**Employment status, n (%)**			
employed 80–100%	55 (16)	21 (12)	34 (21)
employed 50–79%	28 (8)	13 (7)	15 (9)
employed less than 50%	40 (12)	18 (10)	22 (14)
unemployed	216 (64)	126 (71)	90 (56)
**Years since OA diagnosis, n (%)**			
less than 1 year	16 (5)	8 (4)	8 (5)
1 to < 3 years	117 (35)	65 (37)	52 (32)
3 to < 5 years	77 (23)	42 (24)	35 (22)
5 to < 10 years	48 (14)	29 (16)	19 (12)
10 or more years	68 (20)	29 (16)	39 (24)
Don’t know	13 (4)	5 (3)	8 (5)
**Joint(s) with OA, n (%)**			
Hip(s)	61 (18)	28 (16)	33 (20)
Knee(s)	226 (67)	122 (69)	104 (65)
Hip(s) and knee(s)	52 (15)	28 (16)	24 (15)
**Daily living limitation due to OA, n (%)**			
Not at all	36 (11)	17 (10)	19 (12)
Somewhat	261 (77)	139 (78)	122 (76)
Strongly	42 (12)	22 (12)	20 (12)
**Comorbidities, n (%)**			
No comorbidities except knee OA/ hip OA	197 (58)	104 (58)	93 (58)
Diabetes	16 (5)	12 (7)	4 (2)
Cancer	12 (4)	5 (3)	7 (4)
Cardiovascular diseases	43 (13)	21 (12)	22 (14)
Respiratory disease	18 (5)	11 (6)	7 (4)
Musculoskeletal diseases	60 (18)	35 (20)	25 (16)
Others	15 (4)	4 (2)	11 (7)

n (%): absolute and relative frequency

SD: Standard Deviation

OA: Osteoarthritis

### Key findings interviews and focus group

The analysis of the individual interviews and the focus group revealed a category system with barriers, facilitators and support needs as well as more detailed generic categories and subcategories. For the barriers and facilitators four generic categories could be revealed: (1) health- related factors; (2) social factors; (3) personal factors; and (4) environmental factors. More information is provided in the Additional file 2.

### Level of PA and GE adherence

About 84% (*n* = 285) of the respondents met the PA guidelines and stated they performed 150 min per week or more of moderate intensity PA. The recommended GE on two or more days per week was performed by 53% (*n* = 178) respondents according to self-reported data (see Table 2).

**Table 2:** Level of PA and GE adherence

	All participants	GE adherent	GE non-adherent
	(*n* = 339)	*n* = 178 (53%)	*n* = 161 (47%)
PA, (IPAQ-SF)			
Active, n (%)	285 (84)	153(86)	132 (82)
Inactive, n (%)	54 (16)	25 (14)	29 (18)
**Sitting duration n (%)**			
0 to < 3 h/day	40 (12)	19 (11)	21 (13)
≥ 3 to < 6 h/day	158 (47)	95 (53)	63 (39)
≥ 6 to < 9 h/day	70 (21)	34 (19)	36 (22)
≥ 9 to 12 h/day	25 (7)	8 (4)	17 (11)
≥ 12 h/day	46 (14)	22 (12)	24 (15)
**GLA:D exercise**			
Minutes/day, mean (SD)	28 (± 21.7)	35 (± 18.1)	21 (± 23.0)
Adherent, n (%)	178 (53)	178 (100)	
2 days/week, n (%)	96 (28)	96 (54)	
> 2 days/week, n (%)	82 (24)	82 (46)	
Non-adherent, n (%)	161 (47)		161 (100)
0 day/week, n (%)	71 (21)		71 (44)
1 day/week, n (%)	90 (27)		90 (56)

SD: Standard Deviation

n (%): absolute and relative frequency

PA: Physical Activity

GE: GLA:D Exercise

IPAC-SF: International Physical Activity Questionnaire Short-Form

GLA:D: Good Life with osteoArthritis Denmark

Active: ≥ 150 min/week moderate PA

Inactive: < 150 min/week moderate PA

Adherent: performing ≥ 2 times per week GE

Non-adherent: performing < 2 times per week GE

### Barriers and facilitators affecting long-term GE adherence

The respondent’s ratings in the survey of the barriers are shown in Table 3 and the facilitators in Table 4.

**Table 3:** Barrier ratings

		Items rated as rather hindering/very hindering by				
		*n* (%)**				
Barriers	Items rated as not applicable	All	GE adherent	GE non-adherent	OR (95% CI)	*p-value
	*n* = 339	*n* = 339	***n*** ** = 178 (53%)**	***n*** ** = 161 (47%)**		
**Health-related factors**						
Low energy	46 (14)	85 (29)	38 (24)	47 (34)	0.62 (0.36–1.06)	0.07
Pain-free before exercising	40 (12)	44 (15)	19 (12)	25 (18)	0.64 (0.31–1.27)	0.19
Pain before exercising	32 (9)	82 (27)	43 (26)	39 (28)	0.9 (0.52–1.54)	0.70
Pain during or after exercising	35 (10)	81 (27)	38 (23)	43 (31)	0.67 (0.39–1.15)	0.15
Swelling, feeling blockage and/or stiffness	61 (18)	70 (25)	30 (20)	40 (31)	0.56 (0.31–1.01)	**0.04**
No physical limitations in daily life	42 (12)	51 (17)	24 (15)	27 (19)	0.77 (0.4–1.47)	0.44
Reduced general health	57 (17)	74 (26)	42 (27)	32 (25)	1.13 (0.64-2)	0.69
Uncertainty about how GE can positively influence the course of osteoarthritis	79 (23)	27 (10)	14 (10)	13 (11)	0.96 (0.4–2.33)	1.00
Uncertainty about practical GE performance	83 (24)	10 (4)	4 (3)	6 (5)	0.57 (0.12–2.46)	0.52
No exercising before GLA:D programme participation	108 (32)	24 (10)	10 (8)	14 (13)	0.63 (0.24–1.61)	0.39
**GE programme related factors**						
GE programme is boring	51 (15)	38 (13)	13 (9)	25 (18)	0.42 (0.19–0.89)	**0.02**
GE programme takes a long time	42 (12)	73 (25)	28 (18)	45 (32)	0.48 (0.27–0.84)	**0.01**
GE are not individually adapted	67 (20)	23 (8)	15 (10)	8 (6)	1.69 (0.64–4.77)	0.28
GE are difficult to perform	69 (20)	8 (3)	4 (3)	4 (3)	0.78 (0.14–4.3)	0.73
**Social factors**						
No/little support and encouragement from family and/or friends	67 (20)	34 (13)	10 (7)	24 (19)	0.33 (0.13–0.74)	**0.01**
No exercise-partner available	49 (14)	64 (22)	19 (12)	45 (33)	0.28 (0.15–0.53)	**0.00**
No possibility to exercise in a group	50 (15)	62 (21)	23 (15)	39 (29)	0.43 (0.23–0.79)	**0.00**
No relationship of trust between patient and GLA:D physiotherapist	120 (35)	9 (4)	4 (3)	5 (5)	0.63 (0.12–2.99)	0.51
No/little support from GLA:D physiotherapist	121 (36)	17 (8)	3 (3)	14 (14)	0.16 (0.03–0.59)	**0.00**
No/little encouragement from GLA:D physiotherapist	120 (35)	16 (7)	5 (4)	11 (11)	0.34 (0.09–1.1)	0.07
**Personal factors**						
No/little progress and improvements	79 (23)	53 (20)	24 (17)	29 (24)	0.68 (0.35–1.29)	0.22
No/little intention to perform GE	79 (23)	44 (17)	12 (9)	32 (25)	0.3 (0.13–0.63)	**0.00**
No/little motivation to perform GE	64 (19)	91 (33)	24 (18)	67 (49)	0.23 (0.12–0.4)	**0.00**
No/little self-discipline to perform GE	58 (17)	112 (40)	38 (26)	74 (54)	0.31 (0.18–0.52)	**0.00**
Boredom while performing GE	67 (20)	55 (20)	18 (13)	37 (29)	0.35 (0.18–0.68)	**0.00**
No/little confidence to perform GE independently	92 (27)	24 (10)	10 (7)	14 (13)	0.55 (0.21–1.4)	0.20
**Organisational factors**						
No/little time to perform GE	58 (17)	85 (30)	29 (19)	56 (42)	0.33 (0.19–0.58)	**0.00**
Lack of regularity to perform GE	56 (17)	81 (29)	20 (14)	61 (44)	0.2 (0.11–0.37)	**0.00**
No/too little integration of GE into the daily/weekly structure	56 (17)	90 (32)	21 (14)	69 (50)	0.17 (0.09–0.31)	**0.00**
Lack of external pressure (e.g. appointment)	70 (21)	80 (30)	15 (11)	65 (48)	0.14 (0.07–0.26)	**0.00**
Supporting aids for GE not available	92 (27)	16 (6)	5 (4)	11 (9)	0.38 (0.1–1.23)	0.12

n (%): absolute and relative frequency

OR (95%CI): odds ratio with 95% confidence interval (CI)

GE: GLA:D Exercises

*p-value from Fisher’s exact test, comparing the proportion of ratings between adherent vs. non-adherent participants

** All participants selecting the answer option “not applicable” were excluded in the calculation of the relative frequency to minimise distortions

**Table 4:** Facilitators rating

		Items rated as rather facilitating/very facilitating by				
		*n* (%)**				
Facilitators	Items rated as not applicable	All	GE adherent	GE non-adherent	OR (95% CI)	*p-value
	*n* = 339	*n* = 339	***n*** ** = 178 (53%)**	*n* ** = 161 (47%)**		
**Health-related factors**						
High energy	33 (10)	237 (77)	129 (79)	108 (76)	1.23 (0.69–2.18)	0.49
Pain before exercising	58 (17)	125 (44)	61 (41)	64 (48)	0.74 (0.45–1.21)	0.23
Pain-free before exercising	39 (12)	195 (65)	116 (74)	79 (55)	2.38 (1.43-4)	**0.00**
Pain-free during or after exercising	36 (11)	216 (71)	119 (74)	97 (68)	1.38 (0.81–2.34)	0.25
No swelling, no feeling of blockage or stiffness	51 (15)	207 (72)	118 (77)	89 (66)	1.65 (0.96–2.88)	0.07
Physical limitations in daily life	57 (17)	147 (52)	78 (53)	69 (51)	1.05 (0.64–1.72)	0.91
Good general health	29 (9)	238 (77)	134 (80)	104 (73)	1.52 (0.87–2.68)	0.14
Clarity about how GE can positively influence the course of osteoarthritis	20 (6)	271 (85)	148 (86)	123 (84)	1.2 (0.62–2.33)	0.64
Clarity about practical GE performance	18 (5)	271 (84)	148 (86)	123 (83)	1.2 (0.63–2.31)	0.64
Exercising before GLA:D programme participation	76 (22)	194 (74)	106 (75)	88 (73)	1.1 (0.61–1.99)	0.78
**GE programme-related factors**						
GE programme is varied	26 (8)	266 (85)	148 (86)	118 (84)	1.2 (0.61–2.35)	0.63
Appropriate duration of the GE programme	21 (6)	275 (86)	159 (91)	116 (81)	2.55 (1.25–5.39)	**0.01**
GE are individually adapted	20 (6)	287 (90)	156 (90)	131 (90)	0.93 (0.41–2.06)	0.85
GE are easy to perform	17 (5)	300 (93)	166 (95)	134 (91)	1.79 (0.68–4.89)	0.27
**Social factors**						
Support and encouragement from family and/or friends	65 (19)	195 (71)	107 (73)	88 (69)	1.25 (0.71–2.18)	0.43
Exercise-partner available	111 (33)	128 (56)	60 (52)	68 (60)	0.72 (0.41–1.26)	0.23
Possibility to exercise in a group	95 (28)	149 (61)	69 (56)	80 (67)	0.63 (0.36–1.09)	0.09
Relationship of trust between patient and GLA:D physiotherapist	71 (21)	234 (87)	121 (86)	113 (88)	0.85 (0.38–1.85)	0.72
Support from GLA:D physiotherapist	77 (23)	225 (86)	115 (85)	110 (87)	0.8 (0.37–1.7)	0.60
Encouragement from GLA:D physiotherapist	77 (23)	222 (85)	113 (82)	109 (87)	0.69 (0.33–1.44)	0.31
**Personal factors**						
Progress and improvements	13 (4)	303 (93)	167 (94)	136 (91)	1.59 (0.62–4.2)	0.29
Intention to perform GE	14 (4)	288 (89)	165 (93)	123 (84)	2.47 (1.16–5.51)	**0.01**
Motivation to perform GE	14 (4)	285 (88)	161 (91)	124 (84)	1.94 (0.94–4.1)	0.06
Self-discipline to perform GE	15 (4)	272 (84)	155 (88)	117 (79)	1.95 (1.03–3.77)	**0.03**
Fun while performing GE	20 (6)	263 (82)	145 (84)	118 (80)	1.32 (0.71–2.46)	0.38
Confidence to perform GE independently	18 (5)	284 (88)	161 (91)	123 (85)	1.92 (0.91–4.15)	0.08
**Organisational factors**						
Enough time to perform GE	20 (6)	286 (90)	157 (90)	129 (90)	1.01 (0.46–2.23)	1.00
Established regularity to perform GE	26 (8)	279 (89)	152 (89)	127 (89)	1.06 (0.49–2.31)	1.00
Good integration of GE into the daily/weekly structure	26 (8)	279 (89)	158 (91)	121 (86)	1.65 (0.76–3.65)	0.2
External pressure (e.g. appointment)	104 (31)	131 (56)	51 (44)	80 (67)	0.38 (0.22–0.67)	**0.00**
Supporting aids for GE available	25 (7)	279 (89)	156 (91)	123 (87)	1.5 (0.7–3.27)	0.28

n (%): absolute and relative frequency

OR (95%CI): odds ratio with 95% confidence interval (CI)

GE: GLA:D Exercises

*p-value from Fisher’s exact test, comparing the proportion of ratings between adherent vs. non-adherent participants

** All participants selecting the answer option “not applicable” were excluded in the calculation of the relative frequency to minimise distortions

The top 3 barriers included: (1) *no/little self-discipline to perform GE* (40%, *n* = 112); (2) *no/little motivation to perform GE* (33%, *n* = 91); and (3) *no/too little integration of GE into the daily/weekly structure* (32%, *n* = 90).

The top 3 facilitators were: (1) *GE are easy to perform* (93%, *n* = 300); (2) *Progress and improvements* (93%, *n* = 303); and (3) *GE are individually adapted* (90%, *n* = 287).

Comparisons of the ratings of barriers and facilitators between the GE-adherent and GE-non-adherent groups are shown in Tables 3 and 4, respectively.

The group comparison indicated differences for the top 3 barrier items. The top 3 barriers for the GE-non-adherent respondents were *no/little self-discipline to perform GE* (54%, *n* = 74), *no/too little integration of GE into the daily/weekly structure* (50%, *n* = 69), and *no/little motivation to perform GE* (49%, *n* = 67). In contrast, the top 3 barriers for the GE-adherent respondents were *reduced general health* (27%, *n* = 42), *pain before exercising* (26%, *n* = 43), *and no/little self-discipline to perform GE* (26%, *n* = 38).

Group comparison of the barriers revealed that respondents in the GE-adherent group rated barrier items as ‘rather or very hindering*’* significantly less often for the following factors: Social factors (4 items); Personal factors (4 items); Organisational factors (4 items); Programme-related factors (2 items); and Health-related factors (1 item) (Table 3).

Group comparison of the facilitators revealed that both groups rated the two items *progress and improvements* (94%, *n* = 167 vs. 91%, *n* = 136) and *GE are easy to perform* (95%, *n* = 166 vs. 91%, *n* = 134) as their top 2 facilitators. The other facilitator items differed between the two groups. The adherent respondents rated the facilitators *intention to perform GE* (93%, *n* = 165) and *confidence to perform GE independently* (91%, *n* = 161) high, while the non-adherent respondents rated *GE are individually adapted* (90%, *n* = 156) and *enough time to perform GE* (90%, *n* = 157) high.

Group comparison of the facilitators revealed that respondents in the GE-adherent group rated facilitator items as ‘rather or very facilitating’ significantly less often for the following factors: Personal factors (2 items); Health-related factors (1 item); GE programme-related factors (1 item); and Organisational factors (1 item) (Table 4).

### Support needs

Table 5 details the respondents ratings of the perceived usefulness of the support needs in promoting long-term adherence to GE with the goal of ‘twice a week GLA:D with long-term continuation’. The items were ranked based on their perceived usefulness (‘rather/’very useful’). The top 3 useful support needs were the items: (1) *shortened version (max. 30 min.) of the GE home programme* (75%, *n* = 255); (2) *monthly continuation of small GE groups with GLA:D physiotherapist supervision* (65%, *n* = 221); and (3) *regular testing of individual progress with GLA:D physiotherapist (e.g. 2x/year)* (65%, *n* = 221).

**Table 5:** Support needs rating

	Items rated as rather useful/very useful by *n* (%)				
Support needs	All	GE adherent	GE non- adherent		
	*n* = 339	*n* = 178 (53%)	*n* = 161 (47%)	OR (95% CI)	*p-value
Shortened version (max. 30 min.) of the GE home programme	255 (75)	128 (72)	127 (79)	0.69 (0.4–1.16)	0.17
Monthly continuation of small GE groups with GLA:D physiotherapist supervision	221 (65)	111 (62)	110 (68)	0.77 (0.48–1.23)	0.26
Regular testing of individual progress with GLA:D physiotherapist (e.g. 2x/year)	221 (65)	124 (70)	97 (60)	1.51 (0.94–2.44)	0.09
Weekly continuation of small GE groups with GLA:D physiotherapist supervision	217 (64)	111 (62)	106 (66)	0.86 (0.54–1.37)	0.57
GE continuation in a fitness center	205 (60)	103 (58)	102 (63)	0.79 (0.5–1.26)	0.32
Independent GE performance with an app with GE videos	179 (53)	106 (60)	73 (45)	1.77 (1.13–2.8)	**0.01**
Group counseling on the topic of ‘regular GE continuation in daily life’	175 (52)	86 (48)	89 (55)	0.76 (0.48–1.19)	0.23
Individual counseling on the topic of ‘regular GE continuation in daily life’	155 (46)	87 (49)	68 (42)	1.31 (0.83–2.06)	0.83
Getting the GE programme in form of a poster	142 (42)	79 (44)	63 (39)	1.24 (0.79–1.96)	0.38
Small online GE groups with GLA:D physiotherapist supervision	134 (40)	74 (42)	60 (37)	1.2 (0.76–1.9)	0.44
Platform for networking with other GLA:D participants	91 (27)	48 (27)	43 (27)	1.01 (0.61–1.69)	1.00

n (%): absolute and relative frequency

OR (95%CI): odds ratio with 95% confidence interval (CI)

*p-value from Fisher’s exact test, comparing the proportion of ratings between adherent vs. non-adherent participants

Both the adherent and the non-adherent respondents rated the item *shortened version (max. 30 min.)* as the top useful support service. Significantly more adherent than non-adherent respondents rated the item *independent GE performance with an app with GE videos* (OR 1.77 (1.13–2.8); *p* < 0.01) as ‘rather/very useful’.

The additional file 4 presents the results of the open question from the survey. The people were asked, what other support needs they would wish to achieve this goal ‘twice a week GE with long term continuation’.

## Discussion

This study aimed to explore the barriers, facilitators and support needs for long-term adherence to GE, as experienced by the respondents. This is the first study to explore former GLA:D participants’ perceptions of the barriers and facilitators affecting their long-term GE adherence. Additionally, this study aimed to investigate respondents’ level of self-reported PA and self-reported adherence to GE between five and 17 months after completion of the GLA:D programme.

### Physical activity and GLA:D exercise adherence

The results showed that 84% (*n* = 285) of respondents reached the recommended level of PA and 53% (*n* = 178) reached the GE adherence goal of exercising at least twice weekly.

A survey among the general Swiss population in 2017 showed a prevalence of recommended self-reported PA of 76% [27]. Comparing these results with international literature, a Swedish study by Sturesdotter et al. showed a prevalence of recommended self-reported PA of 79% for people with knee and hip OA at 12 months after a supported self-management programme [28]. The results of the study by Pisters et al. were comparable with this study, with a prevalence of recommended self-reported PA of 87% at 15 months after a behavioural exercise and activity programme followed by booster sessions in the first year after the programme [29].

Compared to the prevalence of recommended PA (84%, *n* = 285) found in this study, a substantially smaller portion of respondents (53%, *n* = 178) were adherent to the GE recommendations of exercising at least twice weekly. These rates are comparable with a Danish cohort of 10’000 participants (unpublished data). Pisters et al. showed similar self-reported exercise adherence results, with a rate of 59% at 15 months after a behavioural exercise programme with booster sessions in the first year after the programme [29].

Interestingly, the relatively low GE adherence rate (53%, *n* = 178) was not reflected in an overall deterioration in pain and functioning over a one-year period [12]. In contrast, the achieved results were sustained and are comparable in all countries where GLA:D programmes are offered. In our study almost 50% of the people did the GE not at all or once per week. Thus, it is still relevant to know more about barriers and facilitators for effectively supporting GE adherence, as we can’t assume that non-adherent participants can keep the results after programme participation to the same extent as adherent participants.

### Barriers and facilitators affecting GE long-term adherence

The top barrier found to GE adherence was *no/little self-discipline to perform GE* and the top facilitator was *GE are easy to perform*. The group comparison showed substantial differences in the ranking of the top barriers and small variations in the ranking of the top facilitators. This reinforces the need to particularly consider the barriers to achieve long-term adherence and to develop strategies to overcome hindering factors.

In general, it is notable that barriers were perceived less hindering than facilitators were perceived as facilitating. For example, 40%, *n* = 112 rated the top barrier as hindering, whereas 94% rated the top facilitator as facilitating. It can be hypothesised that, in general, the facilitators are perceived as being more meaningful and having greater participant focus compared to the barriers, or that people tend to give a socially desired response rather than an accurate one. Another general pattern can be seen in the ratings of barriers and facilitators in the group comparison. Notably, more non-adherent respondents weighted barrier items as being more hindering, whereas the two groups rated facilitator items as being similarly facilitating. It can therefore be hypothesised that respondents in the non-adherent group perceive obstacles as more hindering.

The dominant three barriers revealed in our study are *no/little self-discipline to perform GE, no/little motivation to perform GE* and *no/little integration of GE into the daily/weekly structure*. Lack of motivation seems to be a strong hindering factor regarding exercise adherence, as it was also the most prominent barrier in the study by Knoop et al. [30]. While adherent respondents considered ‘health-related factors’ to be important barriers, non-adherent respondents weighted the personal and organisational barriers higher. It is important to consider these group differences when planning interventions to enhance long-term exercise adherence after GLA:D. The barriers should be evaluated and addressed individually for each GLA:D participant, both during and after the GLA:D programme. The research by Duong et al. confirms that adherence is always influenced by multiple factors, and they differ between individuals and within an individual over time. To overcome lack of self-discipline and lack of motivation, regular supervision with a booster session or monitoring of progress could improve self-efficacy [31]. Regular exercise engagement depends on a complex interplay of physical, personal, psychological, social and environmental factors, as revealed by the systematic review by Kanavaki et al. [19].

The most highly rated facilitator items in this study are *GE are easy to perform*, *progress and improvements*, and *GE individually adapted.* Our finding on the item ‘*GE are individually adapted’* is supported by the review on knee OA and exercise adherence by Marks, which points out the importance of indivdualised exercise prescriptions [32]. The item ‘progress and improvements’ seems to be a strong facilitator, as in the literature review by Dobson et al. many facilitators were related to reinforcement topics like improvement and positive exercise experience [18]. As the extent of the perceived barriers and facilitators diverged significantly between the two groups, the group differences must be considered when developing strategies to enhance long-term adherence to GE. For example, GE adherence should be monitored during and after the programme, barriers and facilitators should be individually identified and addressed using behavioural change tools.

### Support needs

The following top 3 support needs revealed relevant and interesting options to increase long-term GE adherence: (1) *shortened version (max. 30 min) of the GE home programme;* (2) *monthly continuation of small GE groups with a GLA:D physiotherapist supervision;* and (3) *regular testing of individual progress with a GLA:D physiotherapist*.

The adherent and non-adherent respondents agreed on the top useful support service ‘*shortened version (max. 30 minutes) of the GE home programme’*. Thereafter, the ratings varied slightly between the groups. The adherent respondents rated ‘*independent exercising with videos’* as significantly more useful than the non-adherent respondents. This result could be explained by the findings on the barriers and facilitators in our study, which indicate that lack of intention, motivation and self-discipline, as well as the need for external pressure (e.g. appointment) are barriers to long-term GE adherence. Whilst self-efficacy is considered an important component for self-management among patients with OA to affect PA adherence positively [33], Olsson et al. point out that more on-going support might be needed to maintain self-efficacy after a self-management OA programme [34]. Therefore, it may be worthwhile to analyse the factors that strengthen individual self-efficacy, especially for non-adherent persons.

The systematic review by Cinthuja et al. showed that booster sessions appear to enhance exercise adherence in people with lower limb OA, although only up to the 12 months follow-up [20]. These review findings are consistent with other literature, which suggest that people fail to maintain long-term exercise adherence and stress the importance of the provision of support. According to the review by Marks, long-term monitoring is indicated to encourage exercise adherence [32], which is in line with the participant ratings on useful support needs in this study, such as regular testing and GE group offers. Furthermore, behaviour change techniques (BCTs) such as ‘patient- led goal setting’, ‘self- monitoring of behaviour’ and ‘social support’ demonstrated highest effectiveness ratios to promote PA adherence [35]. Duong et al. emphasise that the implementation of BCT’s, such as booster sessions should be used to improve exercise adherence, which supports the findings of this study [31]. The study by Willett et al. concluded that peoples’ perceived beliefs about their capabilities should be targeted by facilitating psychosocial support and access to resources for PA maintenance post-discharge [36]. Therefore, the focus of the suggested monthly post-GLA:D GE groups should not only be on GE performance, individual adaptation and regular testing, but also on psychosocial support. This could minimise main barriers, combatting lack of motivation and encouraging self-discipline. Participants require strategies and interventions to overcome these barriers and appropriate post-GLA:D programmes should be developed.

### Strengths and limitations

The mixed method approach, which links the qualitative exploration of barriers and facilitators in a purposefully selected sample and the quantitative evaluation of identified factors among the community of GLA:D participants, is a strength of this study. It allowed the exploration of all aspects of the barriers and facilitators to long-term GE adherence.

A limitation of the study is the fact that the questionnaire was not statistically validated before use – although it was pilot tested for comprehensibility and completeness (face validity). Furthermore, our sample displayed a relatively high level of participant education. It has previously been shown that people with a lower level of education are less physically active than people with a higher level of education [37]. What may also limit the generalizability is the finding, that the sample contains mostly unemployed people who might have different barriers than employed people. In addition, the exclusion of non-German speaking participants further limits the general applicability of the study. Furthermore, the high drop-out rate was noticeable in the study. The hypothesis for the relatively high drop out rate is the length of the questionnaire, as drop outs occurred not at one special question but more in the course of the whole questionnaire”.

The self-reported measures of the level of PA and GE adherence should be interpreted with caution, due to possible overestimation through social desirability or recall bias. To reduce overestimation future research should measure PA and GE adherence with electronic monitoring like wearables or apps instead of self-reported questionnaire. The perceived level of PA may not correspond with an objectively measured level of PA [38]. Since participation in this study was voluntary, it is likely that mainly persons with a high interest in PA, GE and the importance of barriers and facilitators for GE, may be represented in the study, thus biasing the results.

Regarding the inclusion timeline the authors had to find a middle way between including a broad sample, that reflects reality, and nevertheless narrowing down the time after GLA:D completion, because of its effect on the adherence rate.

### Practical and research implications

The most important barriers, facilitators and useful support needs revealed in this study should guide the development of strategies to enhance long-term GE performance after GLA:D. Regarding the barriers to long-term GE adherence, the highest positive impact should address the lack of self-discipline and motivation and the introduction of time management and behavioural change tools. The former could be achieved through enabling joyful and rewarding moments while exercising and the latter by providing advice and support for the integration of GE into the daily and weekly structure. In the GLA:D exercise group a number of behaviour change tools are already used e.g. graded tasks, feedback, providing information, peer-modelling and self-monitoring. Regarding the facilitators to long-term GE adherence, it is important to focus during and after the GLA:D programme on individually adapted exercises that are easy to perform and that help participants to prioritise their GE.

The results on support needs suggest that GE at home should take no more than 30 min and that there should be a possibility to attend monthly booster sessions in small groups with a GLA:D physiotherapist. Finally, regular testing to measure progress could considerably enhance long-term GE adherence. Further research should focus on strategies to minimise barriers and empower facilitators, as well as to evaluate their effectiveness on long-term GE adherence.

## Conclusions

The GE adherence rates in this study show that maintaining GE after a GLA:D programme over the long-term is challenging and is influenced by many extrinsic and intrinsic factors. The results demonstrate the requirement for additional support to maintain GE adherence in the post-GLA:D phase. Participants need strategies and interventions to overcome their barriers and to enhance the facilitators. Appropriate post-GLA:D programmes to improve long-term adherence are critical. Barriers and facilitators were rated differently by the GE adherent and non-adherent respondents. Therefore, a patient-centered approach with the consideration of individual goals, abilities, barriers and facilitators and the development of individual behavioural change strategies to minimise the barriers and enhance facilitators could support an improvement in long-term GE adherence. The development of a shortened version of the GLA:D programme (maximum 30 min), the introduction of a post-GLA:D group, and a long-term monitoring with regular testing also appear crucial to maximise long-term GE adherence in former GLA:D participants.

### Electronic supplementary material

Below is the link to the electronic supplementary material.

Supplementary Material 1

## Data Availability

The datasets used and/or analysed during the current study are available from the corresponding author on reasonable request.

## Abbreviations

GE: GLA:D exercise (s)
GLA:D: Good Life with osteoArthritis Denmark
OA: Osteo arthritis
PA: Physical Activity
WHO: World Health Organisation
