# Supplementary material for: Facilitators, barriers and support needs to GLA:D exercise adherence – a mixed method study
Source: BMC Sports Sci Med Rehabil. 2024 Jun 13;16:130. doi: 10.1186/s13102-024-00913-6 (PMC11170889; doi:10.1186/s13102-024-00913-6)
Supplement: Supplementary file 1 — Supplementary Material 1 [file 13102_2024_913_MOESM1_ESM.docx]

# Additional files

## Additional file 1

**Semi-structured interview guide**

| **Topic** | **Main question** | **Sub-questions** |
| --- | --- | --- |
| **Round of introductions** | Please introduce yourself with your name, age and briefly tell something about yourself (e.g. your (former) professional activity, popular leisure activities) | - Age, diagnosis (K/H), gender, place of residence (city/country), period according to GLA:D (1-3mt,4-6mt), professional activity (Yes/No), consent to the transcription of anonymised interviews |
| **Introductory question** | How do you assess your health? | - Do you do something special/active for your health/well-being? - What? - Since when? - How often? - To what extent does this have a positive effect on your health? - To what extent is this related to your illness? - What helps you most in connection with your condition? |
| **Main questions** | How important is physical activity for you? | - What does physical activity mean to you and why? - Would you say that you move a lot in your everyday life? - What are the reasons why they move, what are the reasons why they do nothing at all/ little? - How important has physical activity been in your life so far? |
|  | What do you think about GLA:D exercises? | - Make the GLA:D yourself perform exercises?   If Yes:   - What – how often – how long – when – - Why? - What/who supports them to do the exercises?   If no:   - Why not? - What/who is stopping them? - What kind of offer would they want so that they would rather do the exercises? - What makes it difficult to practice regularly? - What influence could the GLA:D exercises have on people suffering from osteoarthritis? |
|  | To preserve the effect of the GLA:D programme, it is recommended to follow the exercise series twice a week. What helps or could help you achieve and adhere to this level of exercise? What are the most barriers hindering you from performing the exercises regularly? | - If you are sometimes not active to this extent, why is that or can you spontaneously create some barriers to the execution of the GLA:D call exercises? - How do you deal with this yourself? - What would help you overcome these barriers? - How can the GLA:D therapists support you? - Can you spontaneously name some things that will help you to get the GLA:D to perform exercises? - What tips would you give to other people with hip or knee osteoarthritis who have not yet done the GLA:D exercises? |
|  | To strengthen health, it is recommended to exercise for at least 150-300 minutes/week. What helps or could help you achieve and maintain this level of exercise? What are barriers? | - If you are sometimes not active in this mass, why is that or can you spontaneously name some barriers to sufficient exercise? - How do you deal with this yourself? - What would help you overcome these barriers? - How can the GLA:D therapists support you? - Can you spontaneously name some things that will help you maintain a favorable level of exercise? - What tips would you give to other people with hip or knee osteoarthritis who have not yet moved? |
| **Final questions** | What surprised/amazed you about today's discussion? | - What would have to be deepened in individual interviews? - Which topics still need to be worked on further? |
|  | If you review the discussion again, what comes into your mind? | - Which barriers or helpful factors do you personally consider to be the most important? |
|  | What do you think were the important elements of today's discussion? | - Take-home message |

## Additional file 2

**Category system qualitative results**

| **Main category** | **Generic category** | **Subcategory** |
| --- | --- | --- |
| Barriers | Health-related factors | - Tiredness/ lack of energy - Pain/ Swelling - Feelings of blockage, stiffness - Comorbidities - Lack of disease understanding - Fears |
|  | Social factors | - Practice alone (e.g. no fun) - Missing group exercise services |
|  | Personal factors | - Lack of meaningfulness - Too much external pressure - Start difficulty (e.g. after a break) - Lack of improvement, setbacks - Expectations of results - Great effort - Lack of joy, desire, fun - Lack of motivation - Lack of self-discipline |
|  | Environmental factors | - Lack of routine, regularity - Lack of daily/weekly structure - Lack of time - Other commitments - Exercise programme takes too long - Motivation depending on seasons - Lack of post-GLA:D support needs - Restricted access (e.g. fitness) - Work situation - Lack of individuality |

| **Main category** | **Generic category** | **Subcategory** |
| --- | --- | --- |
| Facilitators | Health-related factors | - High level of suffering (e.g. pain) - Pain reduction - Experiences of success, visible progress - Helpful knowledge about exercise adherence behavioural change - Improving the quality of life - Individualised exercise programme |
|  | Social factors | - Therapeutic support - External appointments - Contact with other people - Compare yourself with others - Support from family/friends |
|  | Personal factors | - External pressure - Feeling of self-efficacy - Positive experience/feelings of happiness - Feeling of control over osteoarthritis - Personal objectives - Reward after exercising - Inner urge to move - Meaningfulness in exercise - Joy/Fun/Lust - Taking personal responsibility - Self-discipline - Positive thinking |
|  | Environmental factors | - Motivation through leisure activities/living environment - Routine/regular habit - Training in the fitness center - Supporting aids (e.g. home exercise bike) - Natural environment nearby - Given daily/weekly structure - Low logistical effort |

| **Main category** | **Subcategory** |
| --- | --- |
| Support needs | - Individually customise exercise programme - Continuation of GLA:D individual support - Continuation of the GLA:D group - Continuation of GLA:D in the fitness center - GLA:D refresher lessons - Adherence-promoting advice and knowledge transfer - Social exchange |

## Additional file 3

**Self-administered questionnaire**

**Survey on barriers and facilitators for the independent and regular implementation of GLA:D Exercises**

Welcome and thank you for your willingness to participate in this survey.

**Data protection:** Participation is voluntary. By participating, you agree that the anonymized data may be used as part of my master's thesis. You reserve the right to cancel the survey at any time.

**A: Questions about yourself**

**Please indicate your year of birth.**

Year of birth (e.g. 1985): .........

**Please enter your gender.**

□ Female □ Male □ Diverse

**What is your highest level of education?**

□ Compulsory education not completed

□ Completed compulsory education (Primary/Real/Secondary/District School)

□ Commercial school or technical secondary school (1 year), household apprenticeship year

□ Short apprenticeship

□ Vocational apprenticeship

□ High school diploma, teacher seminar

□ Swiss federal professional diploma, higher technical college

□ University, University of Applied Sciences, University of Teacher Education, Swiss Federal Institute of Technology (ETH)

□ other highest educational qualification (please specify): .........

**Are you currently working?**

□ Yes, 80-100%

□ Yes, 50-79%

□ Yes, less than 50%

□ No

**B: Health-related data**

**Since when do you have the medical diagnosis of osteoarthritis?**

□ less than 1 year

□ 1 to < 3 years

□ 3 to < 5 years

□ 5 to < 10 years

□ 10 or more years

□ don't know

**Which joint or joints have you been diagnosed with osteoarthritis?**

□ hip joint(s)

□ knee joint(s)

□ hip and knee joint(s)

**How severely are you limited by hip and/or knee osteoarthritis in your everyday life?**

□ not at all

□ something

□ strong

**Have you completed the GLA:D programme in 2021?**

□ No

Yes - in what period?

□ January to March

□ April to June

□ July to September

□ October to December

**In addition to your hip and/or knee osteoarthritis, do you have one or more of the following conditions?**

*Please tick all the appropriate boxes.*

Yes:

□ diabetes

□ cancer

□ cardiovascular diseases

□ respiratory diseases

□ musculoskeletal diseases (diseases of the musculoskeletal system)

□ other: .........

□ No

**C: Questions about your general physical activity (adapted from IPAQ-SHORT)**

We are interested in finding out, how often and in which intensity you are physically active in your everyday life. The survey refers to the **last 7 days.** Please take into account all physical activities in the context of your work, in the household and garden, to get from one place to another and in your free time for recreation, exercise activities and sports.

The following questions relate to all your **vigorous** and **moderate** physical activities **over the last 7 days. Vigorous** physical activities refer to activities that take hard physical effort and make you breathe much harder than normal and sweat a bit. Moderate physical activities refer to activities in which you breathe a little more than normal, but can still speak.

1. Think about all vigorous physical activities you did for at least 10 minutes without interruption. On how many of the last 7 days have you performed vigorous physical activities such as intense aerobics, jogging, sporty cycling or fast swimming? Please specify the number of days per week.

**.........Days a week**

1. How much time did you usually spend doing vigorous physical activities? Please enter the average per day in minutes.

.........**Minutes per day**

1. Think of the moderate physical activities you performed for *at least 10 minutes* without interruption. On how many of the last 7 days have you performed moderate physical activities, such as lifting or carrying light loads, normal cycling, climbing stairs, hiking or swimming? This does not include walking. Please specify the number of days per week.

**.........Days a week**

1. How much time did you usually spend doing moderate physical activities? Please specify the average per day in minutes.

.........**Minutes per day**

1. On how many of the last 7 days did you walk *for at least 10 minutes* without interruption? This includes walking to get from one place to another, as well as everything else walking for recreation, exercise or leisure. Please specify the number of days per week

**........Days per week**

1. How much time did you usually spend walking on one of those days? Please specify the average per day in minutes.

.........**Minutes per day**

1. How much time did you spend sitting on a week day during the last 7 days? This can include time such as sitting at a desk, visiting friends, sitting or lying in front of the TV, and also sitting on public transport. Please indicate the average seat duration in hours.

......... **Hours per day**

**D. Questions about the GLA:D exercises**

**How many days a week do you perform GLA:D exercises?**

Days a week: ......

**How long do you perform GLA:D exercises on average on such a day?**

Minutes per day: ........

**E. Barriers and support factors for the independent and regular implementation of the GLA:D exercises**

In your GLA:D course, you were informed about the importance of independently performing the GLA:D exercises for the self-management in knee and hip osteoarthritis. Performing GLA:D exercises twice a week has shown good results in research in terms of pain, everyday physical function, walking ability and quality of life. To know more about barriers and facilitators of the independent and regular (2x/week) performance of the GLA:D exercises, we ask you to answer the following questions.

On the following pages please assess the extent to which various aspects are barriers or facilitators for the independent and regular performance of the GLA:D exercises. Please answer regardless of whether you are currently performing the GLA:D exercises or not.

**Barriers**

**How hindering are the following factors for you to perform GLA:D exercises independently and regularly?**

1 = not hindering at all

2= little hindering

3= rather hindering

4= very hindering

5: not applicable

Thank you for assessing the hindering factors in the previous section of the questionnaire. Since many factors are both hindering and facilitating to the independent and regular implementation of the GLA:D exercises, I would like to ask you to assess in the next section, which factors are facilitating for you to perform the GLA:D exercises independently and regularly.

**Facilitators**

**How facilitating are the following factors for you to perform GLA:D exercises independently and regularly?**

1 = not facilitating at all

2= little facilitating

3= rather facilitating

4= very facilitating

5: not applicable

**Health-related factors**

| **Barriers** | **Facilitators** |
| --- | --- |
| 1. Low energy | High energy |
| 2. Pain free before exercising | Pain before exercising |
| 3. Pain before exercising | Pain free before exercising |
| 4. Pain during or after exercising | Pain free during or after exercising |
| 5. Swelling, feeling blockage and/or stiffness | No swelling, no feeling of blockage or stiffness |
| 6. No physical limitations in daily life | physical limitations in daily life |
| 7. Reduced general health | Good general health |
| 8. Uncertainty about how GE can positively influence the course of osteoarthritis | Clarity about how GE can positively influence the course of osteoarthritis |
| 9. Uncertainty about practical GE performance | Clarity about practical GE performance |
| 10. No exercising before GLA:D programme participation | Exercising before GLA:D programme participation |

**GLA:D programme-related factors**

| **Barriers** | **Facilitators** |
| --- | --- |
| 11. GE programme is boring | GE programme is varied |
| 12. GE programme takes a long time | Appropriate duration of the GE programme |
| 13. GE are not individually adapted | GE are individually adapted |
| 14. GE are difficult to perform | GE are easy to perform |

**Social factors**

| **Barriers** | **Facilitators** |
| --- | --- |
| 15 No/little support and encouragement from family and/or friends | Support and encouragement from family and/or friends |
| 16. No exercise partner available | Exercise partner available |
| 17. No possibility to exercise in a group | Possibility to exercise in a group |
| 18. No relationship of trust between patient and GLA:D physiotherapist | Relationship of trust between patient and GLA:D physiotherapist |
| 19. No/little support from GLA:D physiotherapist | Support from GLA:D physiotherapist |
| 20. No/little encouragement from GLA:D physiotherapist | Encouragement from GLA:D physiotherapist |

**Personal factors**

| **Barriers** | **Facilitators** |
| --- | --- |
| 21. No/little progress and improvements | Progress and improvements |
| 22. No/little intention to perform GE | Intention to perform GE |
| 23. No/little motivation to perform GE | Motivation to perform GE |
| 24. No/little self-discipline to perform GE | Self-discipline to perform GE |
| 25. Boredom while performing GE | Fun while performing GE |
| 26. No/little confidence to perform GE independently | Confidence to perform GE independently |

**Organizational factors**

| **Barriers** | **Facilitators** |
| --- | --- |
| 27. No/little time to perform GE | Enough time to perform GE |
| 28. Lack of regularity to perform GE | Established regularity to perform GE |
| 29. Not/too little integration of GE into the daily/weekly structure | Good integration of GE into the daily/weekly structure |
| 30. Lack of external pressure (e.g. appointment) | External pressure (e.g. appointment) |
| 31. Supporting aids for GE not available | Supporting aids for GE available |

**F. Questions to useful support needs post GLA:D**

After you have answered the questions about the barriers and facilitators for independent and regular GLA:D exercise, we are interested how we could support former GLA:D participants to perform GE independently and regularly and continue it in the long term. To maintain the effect of the GLAD programme on osteoarthritis, it is recommended to perform GE twice a week.

**How useful do you consider the following support needs to achieve this goal 'twice a week GLA:D with long term continuation?**

0= not useful at all

1= little useful

2= rather useful

3= very useful

**Post-GLA:D programme services**

□ Weekly continuation of small GE groups with GLA:D physiotherapist supervision

□ Monthly continuation of small GE groups with GLA:D physiotherapist supervision

□ Small online GE groups with GLA:D physiotherapist supervision

□ Independent GE performance with an app with GE videos

□ GE continuation in a fitness center

□ Shortened version (max. 30 min.) of the GE home programme

□ Getting the GE programme in form of a poster

□ Regular testing of individual progress with GLA:D Physiotherapist (e.g. 2x/year)

**Organizational support**

□ Platform for networking with other GLA:D participants

**Post-GLA:D programme counseling**

□ Group counseling on the topic of 'regular GE continuation in daily life’

□ Individual counseling on the topic of 'regular GE continuation in daily life’

**What other support needs would you wish, to achieve this goal 'twice a week GE with long term continuation?**

**………………………………………………………………………………………………………**

**Thanks for your participation in the survey**

## Additional file 4

**Summary of comments regarding post-GLAD programs**

| **Total comments n (%)  104 (31)** | |
| --- | --- |
| **Main topics, n (%)** | **Comments** |
| **A. GLA:D satisfaction, dissatisfaction, other topics, 52(50)** | |
| **B. Support services, 52(50)** | |
| Monthly/ weekly continuation of small GE groups with GLA:D physiotherapist supervision,  11 (11) | - Regular exercise control - Avoid exercise mistakes - External pressure helps for more motivation |
| Regular testing of individual progress, coaching, exercise control with GLA:D Physiotherapist (e.g. 2x/year), 9 (9) | - Helpful pressure to perform GE regularly - Give feedback about progress and how to perform the exercises more vigorous - Measure individual progress for more motivation - Exercise adaption and control (intensity) |
| GE continuation in a fitness center, 7 (7) | - Helpful supervision (safe exercising) - regular exercise control - Gain knowledge about how to perform GEs in the fitness center on the machines - Combination GE program with/without machines |
| Regular GLA:D newsletter, 4 (4) | - Send a motivational message to enhance the exercise motivation - Inform about the latest osteoarthritis scientific findings and changes in the GLA:D program |
| More individualized GE program,  4 (4) | - Individualized GE (pain adapted) - Work with pain specialized therapist |
| Support the coordination of an unsupervised post GLA:D exercise group (network former GLA:D participants) 3 (3) | - Share experiences - Mutual motivation for GE performance - Regular meetings give external pressure |
| App/GE videos 3 (3) | - Daily reminder - Exercise videos |
| Development of GE program, 2 (2) | - Provide more exercise variety to foster motivation |
| Shortened version of the GE program for at home, 2 (2) | - Short version GE , max. 15 minutes - Short version GE, max. 30 minutes |
| Outdoor group training with physiotherapist, 2 (2) | - Monthly outdoor group training (GEs, stairs, Walking) - Development of indoor and outdoor GE program |
| Repetition of the GLA:D program,  2 (2) | - Repetition of the whole GLA:D program (half-yearly) |
| Individual counseling about exercise behavioural change  1 (1) | - get helpful advice to change exercise behaviour |
| Combination GE and yoga/pilates group trainings, 1 (1) | - Sessions with combination GE and yoga/ pilates |
| Aquafit Sessions 1 (1) | - aquafit sessions (daily, weekly) |
